# Correlation between cognitive impairment and serum phosphorylated tau181 protein in patients with preeclampsia

**DOI:** 10.3389/fnagi.2023.1148518

**Published:** 2023-03-28

**Authors:** Yuanyuan Wang, Bin Guo, Ke Zhao, Linfeng Yang, Tao Chen

**Affiliations:** ^1^Department of Radiology, Binzhou Medical University, Yantai, Shandong, China; ^2^Department of Radiology, Jinan Maternity and Child Care Hospital Affiliated to Shandong First Medical University, Jinan, Shandong, China; ^3^Department of Clinical Laboratory, Jinan Maternity and Child Care Hospital Affiliated to Shandong First Medical University, Jinan, Shandong, China

**Keywords:** preeclampsia, cognitive function, tau protein, serum phosphorylated tau181 protein (P-tau181), Symbol Digit Modalities Test (SDMT)

## Abstract

**Objective:**

To study the cognitive function status, serum phosphorylated tau181 (P-tau181) protein level, and total tau (T-tau) protein level in patients with preeclampsia (PE), pregnant healthy controls (PHCs), and non-pregnant healthy controls (NPHCs), and to research their feasibility as serum biomarkers for evaluating cognitive functional impairment in PE patients.

**Methods:**

Sixty-eight patients with PE, 48 NPHCs, and 30 PHCs were included. Cognitive functional status was assessed using standardized Symbol Digit Modalities Test (SDMT) and Montreal Cognitive Assessment (MoCA) scales. Enzyme-linked immunosorbent assay (ELISA) was used to detect the level of serum P-tau181 and T-tau protein. The concentration of serum P-tau181 and T-tau protein were compared by one-way analysis of variance in the three groups of subjects. The correlation between P-tau181, T-tau, and SDMT was explore by multiple linear regression analysis. The areas under the receiver operating characteristic (ROC) curves of serum P-tau181 and SDMT were calculated to predict the cognitive level of subjects.

**Results:**

PE patients significantly had lower scores on SDMT (47.97 ± 7.54) and MoCA (28.00 ± 2.00) than normotensive PHCs (30.00 ± 1.25, 54.73 ± 8.55, respectively). The significant difference was found in serum P-tau181 protein levels among the three groups [*H(K)* = 19.101, *P* < 0.001]. Serum P-tau181 was thicker in PE patients than PHCs or NPHCs (both *P* < 0.05). According to the ROC curve, T-tau had no statistical significance in predicting the ability of cognizance, while P-tau181 and SDMT had. The DeLong test showed that P-tau181 was better than T-tau in predicting the ability of cognizance (*P* < 0.05).

**Conclusion:**

The patients with PE have occurred the decline of cognitive function during pregnancy. The high level of serum P-tau181 can be used as a clinical laboratory indication for non-invasive assessment of cognitive functional impairment in PE patients.

## Introduction

Preeclampsia (PE) is a cumulative multisystem disease, especially of the central nervous system, and defined as hypertension with end-organ dysfunction after 20 weeks of gestation which complicates approximately 4–6% of all pregnancies (Abalos et al., 2013; Chappell et al., 2021). The major causes of severe maternal morbidity and mortality were due to the complications of PE in the brain, including eclampsia, cerebral edema, and stroke (Fishel Bartal and Sibai, 2022). The central nervous system involvement and complications of PE have been recognized for many years, but a good understanding of the long-term consequences of PE, such as cardiovascular and cerebrovascular disease and cognitive impairment, can only be traced back nearly a decade ago (Shawwa et al., 2018).

Brain damage from severe PE is not fully reversible, and PE patients have a higher risk of cognitive impairment, vascular dementia, epilepsy, and stroke than healthy women in the months to years after pregnancy (Nerenberg et al., 2017; Basit et al., 2018). Women with a history of severe PE have poorer learning abilities, more memory problems 3–8 months postpartum, and more working memory problems 6–18 months postpartum (Brussé et al., 2008; Baecke et al., 2009). A recent long-term follow-up study found that women with a history of PE occurred objective cognitive decline after 15 years, more problems with working memory and language learning than previously normotensive women, even clinical depressive symptoms (Adank et al., 2021). In addition, PE can significantly impair the working memory of the patient’s offspring (Rätsep et al., 2016). However, there are few studies on cognitive function changes in PE patients.

Serum phosphorylated tau (P-tau) protein and total tau (T-tau) protein are markers of cognitive impairment and are closely related to the cognitive function of individuals. Tau protein is the most abundant neuron microtubule-associated protein, mainly distributed in neuron axons, and its function is to promote the formation of microtubules and maintain the stability of microtubules (Iqbal et al., 2010). The balance between phosphorylation and dephosphorylation of tau protein is a key regulatory process for maintaining microtubule stability. In pathological conditions, hyperphosphorylation of tau protein can reduce the transport efficiency of nerve cell axons, leading to synaptic degeneration of nerve cells and apoptosis or death of nerve cells, eventually leading to cognitive impairment, which is called tauopathy (Guo et al., 2017). Neuronal excitotoxicity induces tau misfolding, leading to altered tau structure and function, and this structural shift leads to more organized aggregation and neurofibrillary tangles, resulting in the degeneration and loss of neurons (Ballatore et al., 2007; Lebouvier et al., 2009). Therefore, there is important clinical significance to explore the changes of concentration of serum phosphorylated tau181 (P-tau181) and T-tau in PE patients, pregnant healthy controls (PHCs), and non-pregnant healthy controls (NPHCs), and their correlation with cognitive impairment.

In this prospective study, the purpose is to assess whether PE patients experience changes in cognitive function during pregnancy, as well as the changes of serum P-tau181 protein and T-tau protein in PE patients, and to determine whether these two proteins can serve as serum biomarkers for estimating altered cognitive function in PE patients.

## Materials and methods

### Subjects and PE patient assessment

From July 2019 to April 2020, 68 PE patients, 48 NPHCs, and 30 PHCs were included in this study. This study was carried out after the approved by the Ethics Review Committee of Jinan Maternity and Child Care Hospital Affiliated to Shandong First Medical University. All subjects signed written informed consents and clearly understood the experimental procedures. The patients with PE were voluntarily recruited from the inpatients and outpatients in the hospital obstetric department, and none of them had been diagnosed with eclampsia throughout the pregnancy. PHCs were recruited mainly by brochures, online publicity, and placard in the obstetrics and gynecology clinic, and the demographic information of PHCs was matched to the PE patients, such as age, gestational age, etc. PHCs did not include premature infants (<37 weeks) and abnormal weight neonates (reference birth weight ±2 SD for sex and gestational age). The recruitments of the age-appropriate NPHCs was mainly through local community networks. The baseline data of PHCs and NPHCs was collected within 12 h after study enrollment, including underwent blood pressure (BP) measurements, collection of blood and urine sample, neurological examinations, and cognitive function tests within 12 h of study enrollment. Blood pressure values of the right arm were measured after the subjects were placed in a quiet supine position for 15 min. All subjects were right-handed in this experiment and underwent routine blood and urine tests. All NPHC participants had laboratory test results within 7 days.

Preeclampsia patients all meet the diagnostic criteria for PE (American College of Obstetricians and Gynecologists [ACOG], 2019). Diagnostic criteria: when gestational age exceeds 20 weeks, subjects were diagnosed with hypertension [means systolic blood pressure (SBP) >140 mmHg and/or diastolic blood pressure (DBP) >90 mmHg] for the first time and found the evidence of organ damage and/or proteinuria, such as hepatic dysfunction, renal insufficiency, thrombocytopenia, pulmonary edema, or central nervous system symptoms. Singleton pregnancies and the gestational age of NPHCs and PHCs were strictly controlled, ranging from 20 to 41 + 6 weeks. Exclusion criteria: diabetes mellitus (either before or during pregnancy), chronic hypertension, and pre-existing renal disease before pregnancy. Subjects with previous diagnoses of PE and pregnancy-induced hypertension were also ineligible for inclusion criteria in PHCs and NPHCs.

### Laboratory test indicators

All subjects received necessary laboratory tests to assist in the diagnosis of PE and to exclude other diseases. All specimens were collected by professional medical technicians, and not receiving the treatment of magnesium sulfate before collection. Five milliliters of fasting venous blood was drawn, and EDTA-K2 anticoagulation and centrifugation were used to separate serum for the detection of relevant laboratory indicators. The measured indicators included, glycosylated hemoglobin, fasting blood glucose, blood lipids, hepatic function, routine blood, and kidney function. Random urine samples (10 ml) were collected from subjects for qualitative detection of urine protein and urinary microalbumin/creatinine ratio. Urinary protein was qualitatively detected by test-paper colorimetry, and the urinary microalbumin/creatinine ratio was detected by immunoscattering turbidimetry. The 24-h urine protein test requires collecting all the urine of the subjects within 24 h and detecting and calculating the total protein in the 24-h urine.

### Neurological exams and cognitive tests

The detailed neurological examination was performed on all participants. All subjects underwent the Symbol Digit Modalities Test (SDMT) and Montreal Cognitive Assessment (MoCA) to evaluate the cognitive function status. ➀ SDMT was used to evaluate attention. The subjects were required to fill in the symbols corresponding to the numbers in sequence as fast as they could within 90 s, and 1 point was awarded for each correctly filled symbol. ➁ MoCA includes visuospatial structure, executive function, attention, language, memory, calculation, orientation, abstract thinking, and other, multidomain assessments. The total score is 30 points, and scores <26 points are abnormal. It has higher sensitivity for screening patients with light cognitive function impairment.

### Detection of serum P-tau181 and T-tau protein concentrations

In this study, enzyme-linked immunosorbent assay (ELISA) was mainly used to obtain the concentration of serum P-tau181 and T-tau. All specimens were collected and tested by professional medical technicians, and not receiving the treatment of magnesium sulfate before collection. Two milliliters of venous blood was drawn rapidly from each subject, and its serum was separated within 1 h. P-tau181 and T-tau reagents were purchased from Wuhan Saipei Biotechnology Co., Ltd. According to the kit instructions, all procedures of the experiment were carried out seriously. The protein concentration of the experimental samples was the average of three repeated measurements.

### Statistical methods

The software we need for statistical analysis in this study included SPSS 26.0, Windows (version 21.0), MedCalc software, and GraphPad Prism 8. First, the basic descriptive analyses was used to perform the variables of the subjects for PE patients, NPHCs, and PHCs. The continuous numerical data was described as the median ± interquartile range or mean ± standard deviation (SD), and the discrete numerical data was expressed as *n* (%). One-way ANOVA and the two-independent-samples rank sum test were used to compare laboratory test indicators and quantitative clinical data. The ability of P-tau181, T-tau, and SDMT to predict cognitive function was evaluated mainly by the area under the curve (AUC) from the receiver operating characteristic (ROC) curve. The correlation between SDMT and clinical data and laboratory tests indicators was explored by Spearman bivariate correlation analysis. The correlation between P-tau181, T-tau, and SDMT was explored by multiple linear regression analysis.

## Results

### Clinical data and cognitive test results of each group

**FIGURE 1:**
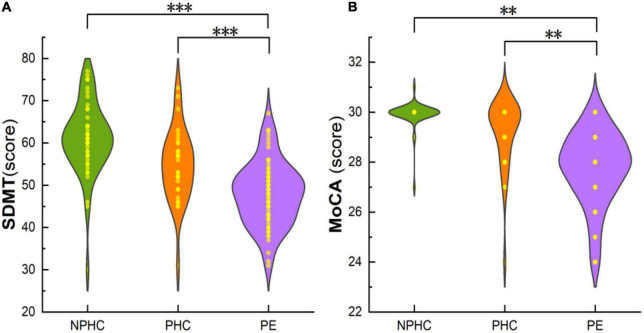
The composite plot of the scatter plot and the smoothed violin plot shows the distribution and frequency of SDMT of subjects in the PE, PHCs, and NPHCs groups **(A)**, and MoCA of subjects in the three groups **(B)**. Significant differences were found in SDMT and MoCA between the three groups [*H(K)* = 56.293, *P* < 0.051; *H(K)* = 77.019, *P* < 0.001]. ^**^P < 0.01; ^***^P < 0.001.

This study included 68 patients in the PE group (average age 31.00 ± 6.75), 30 patients in the PHCs group (average age 30.90 ± 6.08), and 48 patients in the NPHCs group (average age 33.10 ± 4.78). The clinical data and its characteristics of the experimental subjects are shown in Table 1. No significant differences were found in age [*H(k)* = 4.282, *P* = 0.118] or height (*F* = 0.977, *P* = 0.379) among the three groups. No significant differences were found in gestational age (*Z* = −0.154, *P* = 0.877) or pre-pregnancy weight (*Z* = −1.508, *P* = 0.132) between the PE patients and PHCs. Significant differences were found in SBP/DBP, mean atrial pressure, education years, pre-pregnancy BMI, SDMT, and MoCA among the three groups [*H(k)* = 108.932, *P* < 0.001; *H(k)* = 100.438, *P* < 0.001; *H(k)* = 108.338, *P* < 0.001; *H(k)* = 75.519, *P* < 0.001; *F* = 13.898, *P* < 0.001; *H(k)* = 56.293, *P* < 0.001; *H(k)* = 77.019, *P* < 0.001]. The highest values of SBP/DBP, mean atrial pressure, and pre-pregnancy BMI (all *P* < 0.001) were found in the PE group (Table 1). The SDMT and MoCA results are shown in Figure 1.

**TABLE 1:** Clinical characteristics of the pairwise comparison among the three groups.

Features	NPHC (*n* = 48)	PHC (*n* = 30)	PE (*n* = 68)	Statistical value				
				*H(K)*/*F*	*P*	*Post-hoc* test		
						a	b	c
Female (*n*)	48	30	68	NA	NA	NA	NA	NA
Age (years)	33.10 ± 4.78	30.90 ± 6.08	31.00 ± 6.75	4.282^a^	0.118	NA	NA	NA
Gestational age (weeks)	NA	31.31 ± 7.10	33.86 ± 6.36	NA	NA	NA	NA	0.833
Reproductive history (GPL)	NA	G2.00 ± 2.00 P0.00 ± 1.00 L0.00 ± 1.00	G2.00 ± 2.00 P0.00 ± 1.00 L0.00 ± 1.00	NA	NA	NA	NA	NA
Height (cm)	162.50 ± 3.89	161.97 ± 5.24	161.24 ± 5.26	0.977^b^	0.379	NA	NA	NA
Pregnancy weigh (Kg)	NA	63.08 ± 9.11	65.80 ± 15.38	NA	NA	NA	NA	0.132
Weight gain during pregnancy (Kg)	NA	11.38 ± 5.87	13.16 ± 6.21	NA	NA	NA	NA	0.188
Pre-pregnancy BMI (Kg/m^2^)	22.09 ± 2.73	24.06 ± 3.36	26.04 ± 4.87	13.898^b^	<0.001	0.036	<0.001	0.025
Systolic pressure (mmHg)	111.35 ± 9.50	118.63 ± 12.49	156.00 ± 19.50	108.932^a^	<0.001	0.005	<0.001	<0.001
Diastolic pressure (mmHg)	70.00 ± 14.00	75.17 ± 9.53	99.69 ± 10.90	100.438^a^	<0.001	0.003	<0.001	<0.001
Mean atrial pressure (mmHg)	82.44 ± 8.65	89.66 ± 9.80	116.84 ± 15.76	108.338 ^a^	<0.001	0.001	<0.001	<0.001
Education years	16.00 ± 3.00	15.00 ± 4.00	15.00 ± 3.00	75.519^a^	<0.001	<0.001	<0.001	0.008
Symbol Digit Modalities Test (SDMT)	61.00 ± 7.00	54.73 ± 8.55	47.97 ± 7.54	56.293^a^	<0.001	<0.001	<0.001	<0.001
Montreal Cognitive Assessment (MoCA)	30.00 ± 0.00	30.00 ± 1.25	28.00 ± 2.00	77.019^a^	<0.001	<0.001	<0.001	<0.001
**Clinical symptoms**								
Headache	NA	1 (3.33%)	9 (13.24%)	NA	NA	NA	NA	NA
Dizziness	NA	2 (6.67%)	5 (7.35%)	NA	NA	NA	NA	NA
Hypogastralgia	NA	7 (23.33%)	NA	NA	NA	NA	NA	NA
Diminution of vision	NA	NA	4 (5.88%)	NA	NA	NA	NA	NA
Pregnancy complicating primary hypertension	NA	2 (6.67%)	25 (36.76%)	NA	NA	NA	NA	NA
Gestational diabetes mellitus	NA	12 (40.00%)	13 (19.12%)	NA	NA	NA	NA	NA
The thyroid disease	NA	8 (26.67%)	4 (5.88%)	NA	NA	NA	NA	NA
Cardiopathy	NA	2 (6.67%)	4 (5.88%)	NA	NA	NA	NA	NA
Fetal distress in uterus	NA	3 (10.00%)	3 (4.41%)	NA	NA	NA	NA	NA
Fetal growth restriction	NA	1 (3.33%)	12 (2.94%)	NA	NA	NA	NA	NA

Data are mean ± SD or median ± inter-quartile range.

*Post*-*hoc* test: a, NPHC vs. PHC; b, NPHC vs. PE; c, PHC vs. PE. NPHC, non-pregnant healthy control; PHC, pregnant healthy control; PE, pre-eclampsia.

^a^Kruskal–Wallis test, *H(K)*.

^b^One-way ANOVA, *F*.

### Laboratory examinations and P-tau181 and T-tau protein concentrations in each groups

**FIGURE 2:**
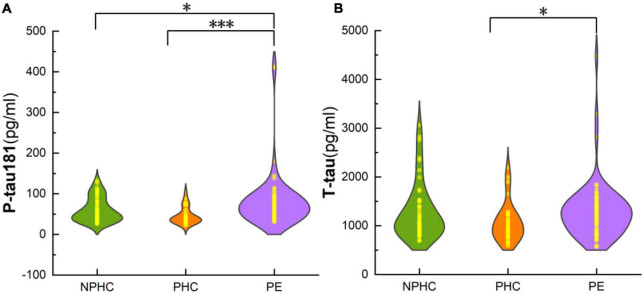
The composite plot of the scatter plot and the smoothed violin plot shows the distribution and frequency of P-tau181 of subjects in NPHCs, PHCs, and PE groups **(A)** and T-tau of subjects in the three groups **(B)**. The significant difference was found in P-tau181 and T-tau among the three groups [*H(K)* = 19.101, *P* < 0.001; *Z* = −2.397, *P* = 0.017]. *P < 0.05; ^***^P < 0.001.

There were significant differences in P-tau181, hemoglobin (Hb), aspartate aminotransferase (AST), and creatinine (Cr) among PE patients, NPHCs, and PHCs groups [*H(K)* = 19.101, *P* < 0.001; *H(K)* = 14.921, *P* = 0.001; *F* = 15.118, *P* = 0.001; *F* = 17.746, *P* < 0.001]. No significant differences was found in T-tau, platelets (Plt), blood glucose (Glu), glycosylated hemoglobin (HbA1c), or alanine aminotransferase (ALT) between the three groups [*H(K)* = 5.937, *P* = 0.051; *F* = 1.324, *P* = 0.269; *F* = 0.275, *P* = 0.871; *F* = 1.649, *P* = 0.438; *F* = 3.273, *P* = 0.195], but a significant difference was found in T-tau between the PHCs and PE groups [*H(K)* = −2.397, *P* = 0.017] (Table 2 and Figure 2).

**TABLE 2:** Laboratory data of the pairwise comparison among the three groups.

Features	NPHC (*n* = 48)	PHC (*n* = 30)	PE (*n* = 68)	Statistical value				
				*H(K)/F*	*P*	*Post hoc* test		
						a	b	c
P-tau181 (pg/ml)	50.17 ± 44.51	37.03 ± 21.50	68.43 ± 47.55	19.101^a^	<0.001	0.028	0.030	<0.001
T-tau (pg/ml)	1,064.12 ± 614.25	969.87 ± 443.51	1,245.72 ± 585.93	5.937^a^	0.051	0.083	0.453	0.017
Hemoglobin (Hb) (g/L)	130.50 ± 13.75	118.97 ± 12.04	124.50 ± 10.75	14.921^a^	0.001	0.001	0.016	0.013
Platelet (Plt) (×10^9^/L)	213.15 ± 49.28	209.43 ± 45.47	209.75 ± 67.50	1.324^b^	0.269	0.754	0.439	0.641
Blood glucose (Glu) (mmol/L)	4.64 ± 0.43	4.75 ± 0.67	4.50 ± 1.10	0.275^b^	0.871	0.354	0.749	0.599
Glycated hemoglobin (HbA1c) (%)	5.44 ± 0.29	4.90 ± 1.10	5.00 ± 0.25	1.649^b^	0.438	0.118	0.167	0.398
Alanine transaminase (ALT) (U/L)	12.00 ± 9.00	12.00 ± 6.00	13.00 ± 15.00	3.273^b^	0.195	0.096	0.955	0.103
Aspartate aminotransferase (AST) (U/L)	16.00 ± 9.00	14.90 ± 4.18	19.00 ± 11.25	15.118^b^	0.001	0.077	0.031	<0.001
Creatinine (Cr) (μmol/L)	47.73 ± 12.04	42.07 ± 9.90	53.00 ± 17.75	17.746^b^	<0.001	0.034	0.024	<0.001
24 h-urine protein (mg)	NA	NA	1,146.00 ± 4,819.03	NA	NA	NA	NA	NA
Albumin/creatinine ratio	NA	0.03 ± 0.39	0.89 ± 3.31	NA	NA	NA	NA	<0.001

Data are mean ± SD or median ± inter-quartile range.

*Post*-*hoc* test: a, NPHC vs. PHC; b, NPHC vs. PE; c, PHC vs. PE. NPHC, non-pregnant healthy control; PHC, pregnant healthy control; PE, pre-eclampsia.

^a^Kruskal–Wallis test, *H(K)*.

^b^One-way ANOVA, *F*.

### ROC curves to evaluate the ability of cognition-predictive in t-tau, P-tau181, and SDMT

**FIGURE 3:**
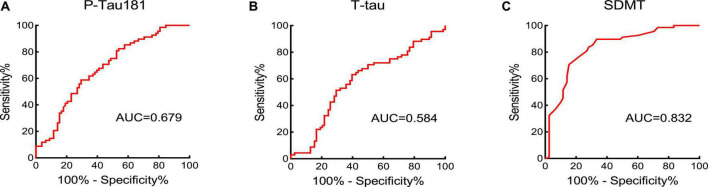
The area under the curve (AUC) value was obtained by receiver operator characteristic (ROC) curve to assess the cognition-predictive ability of P-tau181 **(A)**, T-tau **(B)**, and SDMT **(C)**.

According to the ROC curve, P-tau181 and SDMT can commendably predict the cognitive level of the subjects. The PE group was set as the experimental group, and the NPHC and PHC groups were the control groups. The ROC curves of P-tau181, T-tau, and SDMT are shown in Figure 3. According to the ROC curve, T-tau had no significant ability to predict cognitive ability, while P-tau181 and SDMT had. The DeLong test showed that P-tau181 was better than T-tau in predicting the ability of cognizance (*P* < 0.05) (Figure 3).

### Correlation between P-tau181, T-tau, and SDMT by stepwise multiple linear regression analysis

**FIGURE 4:**
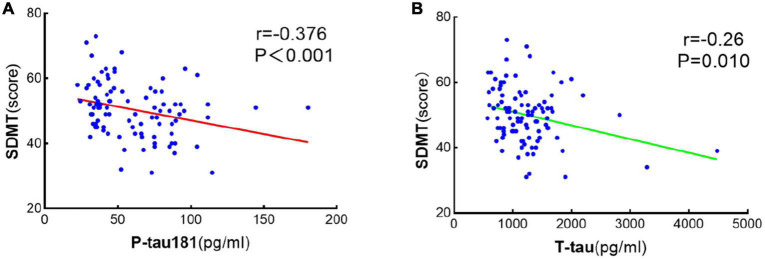
Scatter linear regression plot between SDMT and P-tau181 **(A)** and between SDMT and T-tau **(B)**. It was statistically significant that SDMT was negatively correlated with both P-tau181 and T-tau.

Spearman bivariate correlation analysis found that P-tau181 and mean arterial pressure were positively correlated with SDMT in NPHCs (*r* = 0.196, *P* = 0.183; *r* = 0.115, *P* = 0.435); T-tau, BMI, and age were negatively correlated with SDMT in the NPHCs (*r* = −0.066, *P* = 0.654; *r* = −0.201, *P* = 0.171; *r* = −0.137, *P* = 0.352). P-tau181, T-tau, mean arterial pressure, and BMI were all negatively correlated with SDMT by the multiple linear regression analysis of NPHCs and PHCs (*r* = −0.376, *P* < 0.001; *r* = −0.260, *P* = 0.010; *r* = −0.397, *P* < 0.001; *r* = −0.398, *P* < 0.001) (Table 3). The correlations between T-tau and SDMT as well as between P-tau181 and SDMT are shown in Figure 4. In the multiple linear regression analysis of NPHCs and PHCs, the independent variables of P-tau181, mean arterial pressure, and BMI were all correlated with SDMT. The analysis of collinear effects was performed by the variance inflation factor (VIF) and tolerance in the multiple linear regression analysis. There was no multicollinearity in the model, since the VIFs of all dependent variables were less than 10 (Table 4). The SDMT value was significantly correlated with P-tau181 in the final multivariate analysis (*r* = −0.376, *P* ≤ 0.001) (Figure 4).

**TABLE 3:** Bivariate correlation analysis between the SDMT and the clinical and laboratory data.

	NPHC		Pregnant women	
	*r*	*P*	*r*	*P*
P-tau181 – SDMT	0.196^b^	0.183	-0.376^a^	<0.001
T-tau – SDMT	-0.066^b^	0.654	-0.260^a^	0.010
Mean arterial pressure – SDMT	0.115^b^	0.435	-0.397^a^	<0.001
Body mass index – SDMT	-0.201^b^	0.171	-0.398^a^	<0.001
Age – SDMT	-0.137	0.352	-0.093^a^	0.364
Gestational age – SDMT	−	−	-0.076^a^	0.459

NPHC, non-pregnant healthy control; pregnant women, including PHCs and PE patients; SDMT, Symbol Digit Modalities Test.

^a^Spearman bivariate correlation.

^b^Pearson bivariate correlation.

**TABLE 4:** Linear regression analysis regarding SDMT in pregnant women (*n* = 98).

	Bate	*t*	*P*	Collinearity statistics	
				Tolerance	VIF
Constant	−	14.804	<0.001	−	−
P-tau181 (pg/ml)	-0.193	-2.062	0.042	0.911	1.098
Mean arterial pressure (mmHg)	-0.219	-1.990	0.049	0.656	1.524
Body mass index (Kg/m^2^)	-0.243	-2.165	0.033	0.633	1.580

Pregnant women, including PHCs and PE patients; VIF, variance inflation factor.

## Discussion

In this study, serum P-tau181 and T-tau were detected in PE patients, NPHCs, and PHCs, and tests of cognitive function, including SDMT and MoCA, were performed. The results showed that PE patients had a certain degree of cognitive impairment, as the MoCA and SDMT scores of PE patients were lower than those of the PHCs and the NPHCs. At the same time, the concentration of serum P-tau181 and T-tau were thicker in PE patients than those in PHCs or NPHCs, and the concentration of serum P-tau181 had a great correlation with SDMT. We speculate that P-tau181 protein may lead to cognitive impairment and subsequent neurological symptoms in PE patients, and may serve as a serum biomarker for the diagnosing cognitive functional impairment of PE. According to our investigation, our study is the first to explore the correlation of serum P-tau181 protein level with cognitive impairment in PE patients, NPHCs, and PHCs.

Previous studies have confirmed that the patients with PE have acute or long-term cognitive functional impairment. It is an extremely serious consequence that PE with pulmonary edema or eclampsia can contribute to acute cognitive functional impairment (Bergman et al., 2021). The neurological negative effects of PE over long periods and its complications can lead to an increased risk of seizures, leukodystrophy, vascular dementia, and stroke at older ages (Nerenberg et al., 2017; Basit et al., 2018), but there are few studies on cognitive function changes in PE patients during pregnancy. In this study, detailed neurological examinations and scaled cognitive function tests were performed on all subjects. The symptoms of headache (13.24%), dizziness (7.35%), and vision loss (5.88%) in PE patients were more frequent than those in PHCs. The PE patients had cognitive function scores (SDMT, 47.97 ± 7.54; MoCA, 28.00 ± 2.00) significantly lower than those of normotensive PHCs (54.73 ± 8.55, 30.00 ± 1.25, respectively), indicating that the cognitive function and information processing speed of PE patients had decreased. Although the MoCA scores in PE patients were all higher than the recommended threshold for cognitive impairment (<26 points), the subjects in this group had indeed experienced a decline in cognitive function, which warrants attention from clinicians. Bergman et al. (2021) suggested that PE patients had lower MoCA scores than normotensive pregnant women when they presented with symptoms of eclampsia or pulmonary edema, while they found no difference in MoCA score between PE women without severe features and normotensive women. Our study found that PE patients had already developed cognitive impairment before severe complications.

The mechanism of cognitive impairment in PE has been studied. PE is a multisystem disease that affects multiple organ systems, including the maternal brain. Some hormones secreted through the placenta, such as sex steroids, estradiol, and progesterone, are all markedly elevated during pregnancy and act on neuronal populations through the blood-brain barrier *via* receptors expressed in specific regions (Blennow and Zetterberg, 2018). The impairment of the autoregulation of cerebral circulation in patients with PE leads to the increase of blood–brain barrier permeability and the change of cerebral blood flow (Bergman et al., 2019). Tau protein is one of the main components of neuronal microtubule-associated proteins, and the expression and modification function of tau protein are closely related to cognitive function, and also can reflect the function of neuronal cells. In addition, some scholars have hypothesized that pregnancy may serve as a stress test that can reveal a woman’s risk of developing cardiovascular disease in later life, due to PE shares common risk factors with cardiovascular disease and dementia (Williams, 2003).

In this study, we endeavored to explore serum biomarkers that could assess cognitive impairment in PE patients. Many laboratory indicators can be used to diagnose PE, such as placental growth factor (PlGF), soluble endoglin (sEng), and soluble vascular endothelial growth factor receptor 1 (sFlt-1). These indicators can reflect the oxidative stress of the placenta and the functional status of the vascular endothelial cells, and have good predictive value for PE (Zeisler et al., 2016), but cannot reflect the cognitive function status of PE patients. P-tau and T-tau are markers of cognitive impairment and are closely related to the cognitive function of individuals. P-tau181 is phosphorylated at threonine-181, which is an intermediate product produced during the phosphorylation of tau protein. P-tau181 concentration is associated with cognitive decline (Thijssen et al., 2020; Simrén et al., 2021). By measuring the serum T-tau and P-tau181 concentrations of our subjects, we found that they were both significantly higher in PE patients than PHCs or NPHCs. Lederer et al. (2016) found that the concentration of P-tau181 protein in cerebrospinal fluid was significantly associated with PE/HELLP syndrome (*P* = 0.043: hazard ratio = 1.211). Another report confirmed that the plasma tau protein concentration of women with PE was 2.17 times thicker than that of normotensive women (95% CI, 1.49–3.16). The above results are all in line with the results of our study.

The AUC from the ROC curve analysis have evaluated that P-tau181 had great potential ability of diagnosing cognitive functional impairment in PE patients. Its AUC was 0.679, the cut-off value was 60.95 pg/ml, and P-tau181 performed better than T-tau. Therefore, we believe that the concentration of serum P-tau181 in PE patients is related to cognitive functional impairment and can be used to screen the condition of PE patients. The high level of P-tau181 can be used as a clinical laboratory indication for non-invasive assessment of cognitive functional impairment in PE patients. The P-tau181 cut-off value found in this study must be validated by further studies, which should take into account differences in results caused by different testing methods.

We also studied the correlations between serum P-tau181 and T-tau and the results of cognitive test scales. SDMT can reflect the information processing speed of subjects through attention, visual scanning, and movement speed (Sheridan et al., 2006). In a Parkinson’s disease study, the SDMT and MoCA scales were considered appropriate tools for identifying cognitive features regulated by amyloid-β (Aβ) and tau protein phosphorylation in established synucleinopathies (Fiorenzato et al., 2018). Fiorenzato et al. (2018) found that SDMT scores were significantly lower in Aβ-positive Parkinson’s patients and were significantly associated with increased amyloid deposition in cortical regions (i.e., frontal, posterior cingulate, temporal, parietal, and occipital lobes), which is critical for information processing speed. Aβ can directly induce tau hyperphosphorylation and neurodegeneration (Jin et al., 2011). In our subjects, SDMT was negatively correlated with the serum P-tau181 concentration (*r* = −0.376, *P* ≤ 0.001) in pregnant women at an average gestational age of 33.86 ± 7.46 weeks. However, there was no significant correlation between the two in the NPHCs. Therefore, we believe that the information processing speed can be well reflected by the measurement of serum P-tau181 in women in the third trimester.

Tau and P-tau can be measured by positron-emission tomography (PET) or cerebrospinal fluid (Schöll et al., 2016; Blennow and Zetterberg, 2018), but these methods are invasive and expensive and require high doses of radiation, so they are not suitable for research in pregnant women. We reviewed the literature and found that serum P-tau181 and T-tau protein concentrations both had good correlations with their cerebrospinal fluid protein concentrations (Barthélemy et al., 2020; Janelidze et al., 2020) and can be used as serum biomarkers of cognitive impairment in PE patients. Studies in Alzheimer’s disease confirm the high accuracy of blood P-tau181 assays for identifying individual and combined neurofibrillary tangles and plaque pathology, making it an ideal biomarker for Alzheimer’s disease (Jack et al., 2018).

Subjects in this study could only undergo neuropsychological testing and sample collection when they were clinically stable, and patients with severe neurological symptoms could not participate in this study. Many women with PE are treated with magnesium sulfate during hospitalization to protect their nerves. Clinical practice has confirmed that magnesium sulfate has a favorable effect on improving the cognitive function of pregnant women (Rana et al., 2006). Therefore, neuropsychological testing and sample collection of the subjects should be performed before the use of magnesium sulfate to guarantee the accuracy of the results. Our study was a strictly *in vivo* study based on clinical laboratorial data, but absent from the corresponding pathological evidence of PE. This study has the limitation of small sample size, so it is critically urgent to increase the sample volume to further explore the potential mechanism of cognitive dysfunction in PE patients in the future. This was a preliminary study on serum P-tau181 protein concentration in PE patients. In the future, we will conduct long-term follow-up study of the cognitive function, brain functional imaging characteristics, and related serum markers in PE patients, what is more, further explore the changes of neuroimaging characteristics and serum markers in patients with different severity of cognitive dysfunction. The purpose of our research is to commit to further excavate the pathophysiological mechanisms of PE.

## Conclusion

The serum P-tau181 protein concentration of the PE patients was significantly thicker than that of the PHCs and NPHCs, and had a great correlation with the SDMT and MoCA scores. The results of this study support the potential value of serum P-tau181 protein concentrations in the diagnosis of cognitive functional impairment in PE patients. Serum P-tau181 protein can serve as a simple, accessible, and scalable marker for the screening and diagnosis of cognitive functional impairment in PE.

## Data availability statement

The original contributions presented in this study are included in the article/supplementary material, further inquiries can be directed to the corresponding author.

## Ethics statement

The studies involving human participants were reviewed and approved by the Ethical Committee of the Institutional Review Board (IRB) of Jinan Maternal and Child Care Hospital Affiliated to Shandong First Medical University. The patients/participants provided their written informed consent to participate in this study.

## Author contributions

LY and TC wrote the main manuscript text. BG and KZ prepared the clinical data. YW did the statistical analysis, prepared the figures, and revised the main manuscript text. All authors reviewed the manuscript.
